# Hydraulic and Thermal Performance of Microchannel Heat Sink Inserted with Pin Fins

**DOI:** 10.3390/mi12030245

**Published:** 2021-02-28

**Authors:** Guo-Fu Xie, Lei Zhao, Yuan-Yuan Dong, Yu-Guang Li, Shang-Lin Zhang, Chen Yang

**Affiliations:** 1 Science and Technology on Reactor System Design Technology Laboratory, Nuclear Power Institute of China, Chengdu 610213, China; npichd10@npic.ac.cn (G.-F.X.); zhaolei@npic.ac.cn (L.Z.); dongyuanyuan@npic.ac.cn (Y.-Y.D.); liyuguang@npic.ac.cn (Y.-G.L.); zhangshanglin@npic.ac.cn (S.-L.Z.); 2 Institute of Process Equipment, College of Energy Engineering, Zhejiang University, Hangzhou 310027, China

**Keywords:** microchannel heat sink, pin fin, numerical simulation, hydraulic and thermal performance

## Abstract

With the development of micromachining technologies, a wider use of microchannel heat sink (MCHS) is achieved in many fields, especially for cooling electronic chips. A microchannel with a width of 500 μm and a height of 500 μm is investigated through the numerical simulation method. Pin fins are arranged at an inclined angle of 0°, 30°, 45°, and 60°, when arrangement method includes in-lined pattern and staggered pattern. The effects of inclined angle and arrangement method on flow field and temperature field of MCHSs are studied when Reynolds number ranges from 10 to 300. In addition to this, quantitative analyses of hydraulic and thermal performance are also discussed in this work. With the increase of inclined angle, the variation of friction factor and Nusselt number do not follow certain rules. The best thermal performance is achieved in MCHS with in-lined fines at an inclined angle of 30° accompanied with the largest friction factor. Arrangement method of pin fins plays a less significant role compared with inclined angle from a general view, particularly in the Reynolds number range of 100~300.

## 1. Introduction

The ever-increasing heat flux of microelectronic devices will result in inevitable temperature rise, which poses a great threat to their operation life and promotes the development of cooling technology in microscale as a substitution of conventional cooling method. The discrepancy between thermal expansion coefficients of different parts may bring about undesirable thermal stress [1]. The existence of hot spots also has an adverse impact on the reliability of microelectronic devices or even gives rise to thermal breakdown. Statistics indicate that more than half of malfunctions result from problems in thermal management [2]. Microscale heat transfer devices are three-dimensional structures manufactured by means of specific micromachining technologies [3,4,5]. With the satisfactory heat transfer capability, compact structure, and relatively light weight, the microchannel heat sink (MCHS) can be regarded as a suitable and feasible solution to meet the heat dissipation requirement [6,7]. 

Considering that fluid leakage or structure failure will cause damage to microelectronic elements, laminar flow of coolant fluid is more appropriate in relative terms, which has higher thermal efficiency and lower pressure drop compared with turbulent flow. Tuckerman et al. [8] firstly proposed the idea of microchannel heat sink which can meet the requirement of compact and effective heat removal. They tried to seek an optimum design in order to realize the minimum thermal resistance and found that heat transfer coefficient altered inversely proportional to the characteristic width for laminar flow. Compact integral heat sinks were fabricated according to relevant parameters, including channel width, wall thickness, channel depth and so on. On the basis of researchers’ pioneering work [9] about flat microchannels, the application of Navier–Stokes equations is proved to be valid for this scale, as well as the negligibility of surface roughness, viscous heating and axial heat transfer. In a gesture to avoid rapid temperature increase along the flow direction, the addition of rough elements in MCHS are widely used, such as dimples [10,11], ribs and grooves [12,13,14,15], fins [16,17], and so on [18]. Among them, micro pin fin heat sink (MPFHS) draws a great deal of attention, which is normally consisted of micro channels with an array of aligned or staggered circular pin fins. Shapes of pin fins include, but are not limited to, circular, square [19], hexagon [20], and herringbone [21].

Hasan et al. [22] put an eye on two-fluid counter flow microchannel heat exchangers (CFMCHE). Apart from channel size, the shape of cross-section also plays a significant role in overall performance of CFMCHE. From the results of numerical simulation, it can be noticed that the circular shape gives the best performance from a comprehensive perspective, compared with square, rectangular, iso-triangular, and trapezoidal cross-sectional shape. Correlations considering Reynolds number, thermal conductivity ratio and heat exchanger volume are also developed as a guidance to predict effectiveness and performance index of a microchannel heat exchanger. A parametrical analysis focused on height over diameter ratio *H*/*D* and Reynolds number was undertaken by Koz et al. [23]. The end wall effects on thermal and hydrodynamic performance were quantified, which was verified to be adversely affected by parameters. Relevant flow mechanisms shed light on the difference between microchannel heat sinks and their macroscopic counterparts, delineating the changing situation of fluidic forces and velocity boundary layer near pin fins in simplified models. Holding a suspicious eye toward conclusions obtained in earlier literature, Shafeie et al. [24] applied computer modeling approach to compare the thermo-hydraulic performance of pin-finned heat sink (PFHS) and optimum equivalent MCHS. The first and foremost condition in the investigation is the same pumping power, which is not taken into account before. Entropy generation results indicate that an optimum simple MCHS has an advantage over MPFHS under the same working condition, especially for higher pumper applications. Higher heat removal by optimum MCHS is detected in the pumping power of 0.5 and 2 W. Kim et al. [25] extended relevant work from uniform heat flux condition to constant wall temperature condition. Both general solution and asymptotic solution for microchannel heat sinks of different aspect ratios and porosities are presented and verified by a comparison with numerical results. Conclusion can be made that thermal resistance under the condition investigated is about 10% less than that for constant heat rate condition. Jeng et al. [26] conducted experiments on the forced water cooling process in a pin-fin heat sink made of aluminum alloy, which is inserted with passage divider. The fluid flows along the meandering path created by the left-and-right S-shape divider or up-and-down S-shape divider. Apart from this, packed brass beads of different particle sizes are added simultaneously to the cooling device. Data show that the highest heat transfer enhancement is achieved when ten-millimeter-high pin fins are configured as 3 × 3 combined with up-and-down S-shape divider and filled with brass beads of 4 mm. For the sake of a better cooling effect, a novel design of double-layered microchannel heat sink (DL-MCHS) was introduced by Vafai et al. [27]. Leng et al. [28] employed a simplified conjugate-gradient algorithm to solve for optimal variables of a three-dimensional DL-MCHS model. Great attention was attached to overall thermal resistance and maximum bottom wall temperature to obtain a multi-objective optimization. Results suggested that larger channel number and faster bottom inlet velocity, together with shorter channel width and lower bottom channel height were beneficial to the objective function with the increase of pumping power. 

Aside from structural parameters of MPFHS, the inclined angle and arrangement method of pin fins also make a difference to the hydraulic and thermal performance of the device. The pin fins are mounted at an inclined angle of 0°, 30°, 45°, and 60° to the *y*-axis (the channel width direction). It is worth noting that the *z*-axis denotes the flow direction while the *x*-axis presents the channel height direction. The arrangement method of pin fins follows the in-lined pattern and staggered pattern. Based on the numerical simulation method, flow field and temperature field are illustrated. Friction factor and Nusselt number, as an indication of hydraulic and thermal performance, respectively, are also calculated to investigate the effect. This work is beneficial for further research on the optimum design of MPFHS.

## 2. Numerical Methods

### 2.1. Geometrical Model

**Figure 1:**
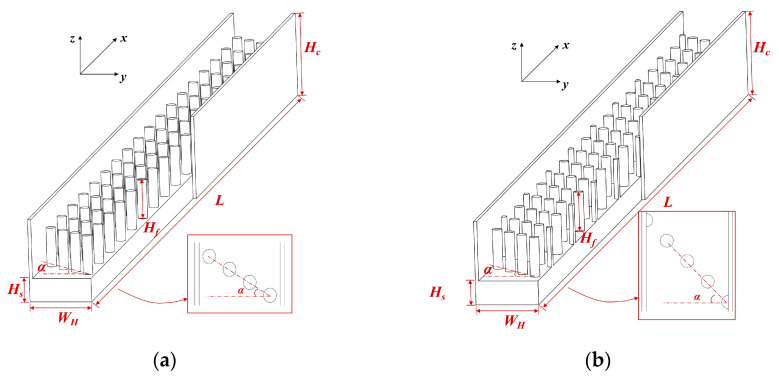
The structure of the investigated MPFHS with: (**a**) in-lined pin fins; (**b**) staggered pin fins.

For the current research, a single channel of MCHS with the inclusion of several typical circular pin fins are investigated. The equivalent diameters of microchannels are usually less than 500 μm, where heat transfer process takes place. As mentioned above, both the channel depth and the channel width exert huge influence on heat removal capacity of MCHS. In addition to this, the arrangement method, cross-sectional shape, and structural parameters of pin fins also play a role in the comprehensive performance of the device. According to reference [24], a channel width of 500 μm, together with a channel height of 500 μm and a fin height of 330 μm is proved to have the highest heat flux during laminar convective heat transfer process in the research scope. Therefore, the channel width *W_H_*, channel height *H_c_* and fin height *H_f_* of the investigated model, as depicted in Figure 1, accord with above. A unit cell of MCHS is selected, and the substrate with a height of *H_s_* is regarded as the heat source. The whole length of the channel *L* is 10 mm, where the first circular pin fin with a diameter of 80 μm is placed 300 μm away from the entrance. The expressions of hydraulic diameter are not consistent in literatures. Referring to Wang’s work [29], it can be calculated as 4*V_f_*/*A_f_*, where *V_f_* and *A_f_* refer to the fluid volume and the wetted surface area of the pin-finned microchannel, respectively. Accordingly, the calculation of Reynolds number can be described as follows:

(1) Re=ρumaxDhμ

where *u_max_* indicates the maximum flow velocity in the fluid region; *ρ* and *μ* indicate density and viscosity, respectively.

The pin fins are arranged oblique to the mainstream direction. There is a need to point out that the inclined angle *α* is defined as the included angle between the orientation of pin fins and the bottom line of the substrate. The investigated inclined angle ranges from 30° to 60°, with an interval of 15°. The pin fins arranged at an inclined angle of 0° is taken as benchmark. From the view of flow, the disposition of pin fins follows the in-lined pattern and staggered pattern, as can be seen in Figure 1a,b, respectively. To put it another way, the numbers of pin fins in the sloping line are inhomogeneous in different arrangement methods. 

The numerical model can be divided as solid part and fluid part. The material of the substrate is silicon, which has a thermal conductivity of 148 W·m^−1^·K^−1^ and a specific heat capacity of 710 J·kg^−1^·K^−1^. The density of silicon is 2330 kg·m^−3^.

### 2.2. Mesh and Boundary Condition

**Figure 2:**
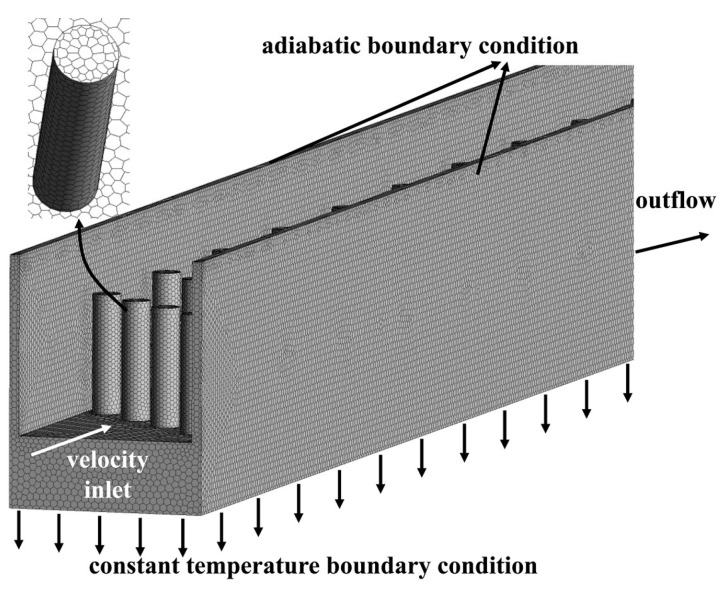
Mesh model and boundary conditions.

Considering that slender channels would increase both the complexity of meshing and the number of meshes, the polyhedral mesh is a desirable selection to discretize the structure. Compared with the hexahedral mesh, it could realize a four-fold reduction in number of cells based on the conclusion of literature [30]. Under the same condition of computational accuracy, the polyhedral mesh is able to reduce the computing time and memory radically. The poly-hexcore mesh generation method contains both hexahedral mesh and polyhedral mesh. The former is adopted for body while the latter for surface, as displayed in Figure 2. 

In addition to the meshing method, calculation precision is also strongly affected by grid size. Three selected sets of grids, which has a cell number of 0.50, 1.05, and 1.90 million, refers to coarse, fine and finer grid condition. When the minimum grid size is controlled to 0.002 mm and the maximum to 0.02 mm, the total number of cells would reach 1,045,658. Following the calculation procedure of the Grid Convergence Index (GCI), the values about heat transfer coefficient are 6.25% and 3.45% for GCI_32_ and GCI_21_. When it comes to pressure drop, the GCI values are 4.04% and 2.20%, respectively. Since both the two comparison indices are in the acceptable range, it can be concluded that the mesh model with a cell number of 1,045,658 is sufficient to obtain accurate data. 

On the assumption that no phase change occurs during the process, an incompressible steady flow is simulated with the tool of ANSYS FLUENT 17.2 (Ansys, Canonsburg, PA, USA), in the Reynolds number range of 10 to 300. There are various options for coolant fluid, among which water is typically preferred by researchers. Subsequently, water is as the fluid flowing through the microchannel, with an initial temperature of 300 K. The thermal conductivity of water is 0.6 W·m^−1^·K^−1^, while the specific heat capacity of 4182 J·kg^−1^·K^−1^. The density of water is 998.2 kg·m^−3^. Additionally, viscosity of water varies with temperature. As seen in Figure 2, the inlet boundary condition is set as velocity inlet, while that for the outlet boundary condition is outflow. The bottom surface of the heat sink is set with a constant temperature of 358 K, which is connected to heat source in reality. Adiabatic boundary condition is imposed to outer walls of the three-dimensional model. No slip and no penetration conditions occur on the interface between solid and fluid. The SIMPLE algorithm is employed for coupling the velocity and pressure fields, while the least square cell-based option for gradient interpolation. Besides, the second order upwind scheme is utilized to complete the discretization of the continuity, momentum and energy equations.

(2) $$\mathrm{The}\;\mathrm{continuity}\;\mathrm{equation}\;\mathrm{for}\;\mathrm{fluid}:\;\;\nabla \cdot \vec{V}=0$$

(3) $$\mathrm{The}\;\mathrm{momentum}\;\mathrm{equation}\;\mathrm{for}\;\mathrm{fluid}:\;\;\rho (\vec{V}\cdot \nabla \vec{V})=-\nabla P+\mu \nabla^{2}\vec{V}$$

(4) $$\mathrm{The}\;\mathrm{energy}\;\mathrm{equation}\;\mathrm{for}\;\mathrm{fluid}:\;\;\;\rho C_{p}\nabla \cdot T_{f}=k_{f}\nabla^{2}T_{f}$$

Accordingly, the energy equation for the solid part can be given as follows:

(5) $$\mathrm{The}\;\mathrm{energy}\;\mathrm{equation}\;\mathrm{for}\;\mathrm{solid}:\;\;\;k_{s}\nabla^{2}T_{s}=0$$

The definition of Fanning’s friction factor is derived from the equation:

(6) $$f=\frac{2\Delta pD_{h}}{\rho Lu^{2}}$$

where *ρ* is the density of water; and *u* is the average inlet velocity.

The local Nusselt number is defined as:

(7) $$Nu_{x}=\frac{h_{i,x}D_{h}}{k_{f}}$$

where *k_f_* refers to the thermal conductivity, W·m_­_^−1^·K^−1^; local heat transfer coefficient *h_i,x_* is determined from Equation (8):

(8) $$h_{i,x}=\frac{q}{T_{s}-T_{f}}$$

where *q* denotes local heat flux.

The average Nusselt number can be calculated from:

(9) $$Nu=\int \frac{Nu_{x}dA}{A}$$

Average thermal resistance of convection *R_conv_* can be a reflection of the thermal performance regarding to the heat sink, which is given by Equation (10):

(10) $$R_{conv}=\frac{T_{a,s}-T_{a,f}}{q^{\prime \prime }{}_{eff}A_{s}}$$

where *T_a,s_* and *T_a,f_* refer to the volume-weighted average temperature of solid part and fluid part, respectively; *A_s_* refers to the total base area of MCHS; $q^{\prime \prime }{}_{eff}$ is the effective input heat flux and its definition is shown as follows:

(11) $$q^{\prime \prime }{}_{eff}=\frac{Q_{fin}}{A_{s}}=\frac{\overset{\cdot }{m}c_{p,f}\left(T_{o}-T_{i}\right)}{A_{s}}$$

where $\overset{\cdot }{m}$ is the mass flux rate of water; *c_p,f_* is specific heat capacity; inlet and outlet temperature of fluid correspond to *T_i_* and *T_o_*, respectively. 

**Figure 3:**
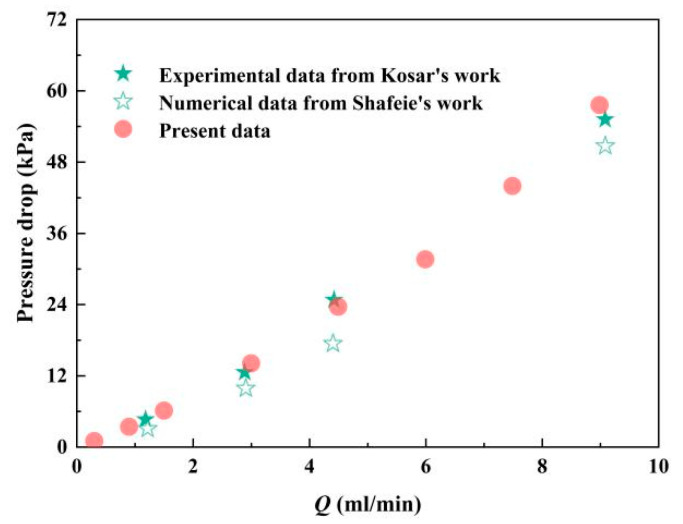
Comparison of present data with Kosar’s work [31] and Shafeie’s work [24].

To verify the reliability of calculated results, the numerical data of present work are compared with experimental data [31] and numerical data [24], which is displayed in Figure 3. The highest error 10.1% is reached with a volumetric flow rate of 9 mL·min^−1^ in comparison with numerical results from Shafeie’s work, which can be calculated as the multiplication of cross-sectional area and velocity. Under the same condition, the error between present work and experimental results is 4.2%, which is within the acceptable range. 

## 3. Results

### 3.1. Hydraulic Performance

**Figure 4:**
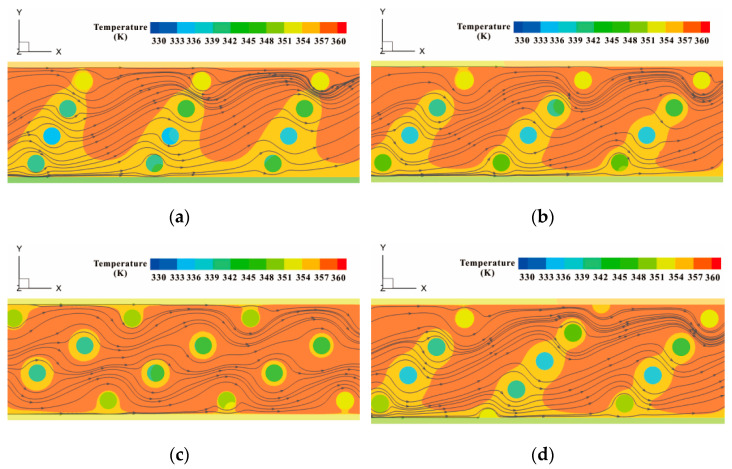
Flow conditions of the MCHS inserting (**a**) in-lined pin fins with an inclined angle of 30°; (**b**) in-lined pin fins with an inclined angle of 45°; (**c**) in-lined pin fins with an inclined angle of 60°; (**d**) staggered pin fins with an inclined angle of 45°.

Due to the presence of pin fins, there are differences between the flow field of diverse MPFHSs. As can be seen in Figure 4a–c, when pin fins are placed at different inclined angles of 30°, 45°, and 60°, disparities emerge between the flowing direction of fluids near pin fins. When bypassing these protrusions, the flow path of fluid deviates from the original route. Some regions become dead zones where few coolant liquid flows through, while more fluid runs through other areas. As a consequence, fluids separate and recirculate in the flowing process, which promotes the mixture of fluids to some extent. Streamlines in four microchannels with pin fins arranged in different patterns are extracted to manifest the trajectory of fluids. Contours in Figure 4 illustrate the temperature of the interface between the substrate and water, at a height of 170 μm along the *z*-axis. The top surface of pin fins can be seen vividly from the perspective of observation, with *x*/*L* ranging from 0.4 to 0.6. Compared with other cases, the temperature of the solid wall with pin fins inclined at an angle of 60° is higher, which can be considered as an indication of worse cooling effect. Comparatively, lower temperature will be obtained when inserting in-lined pin fins with an inclined angle of 30°. In Figure 4b,d, although the layout patterns of pin fins are different, there is some resemblance between the temperature conditions of the interface under the same inclined angle of 45°.

**Figure 5:**
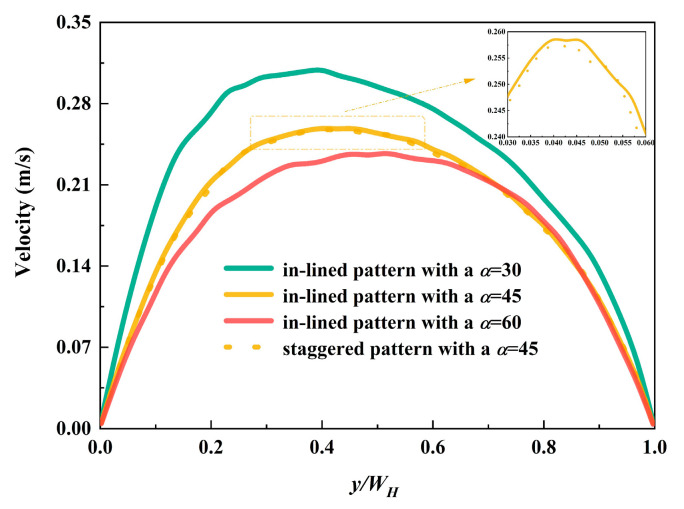
Velocity distribution at the plane of dimensionless flow length *x*/*L* = 0.257.

As the foundation of the following analysis, hydraulic performance plays a crucial role in the evaluation of microchannels. According to the mass conservation equation, the velocity gets accelerated flowing in the limited channels with the presence of pin fins. When inlet velocity is 0.1 m·s^-1^, the velocity conditions at the center line (at a height of *z* = 335 μm) of a certain plane is depicted in Figure 5. Since the distribution modes are analogous excluding the fluid domain near the inlet and outlet regions, only the plane of dimensionless flow length *x*/*L* = 0.257 is chosen. It can be seen from the figure that velocity distribution of the model with in-lined pin fins resembles that with staggered pin fins, provided that the inclined angles are the same. The fastest velocity is over 0.3 m·s^−1^, which occurs in the MCHS with in-lined pin fins arranged at the inclined angle of 30°. The velocity of fluid in the case with *α* = 60° is slower than other conditions, the maximum of which could reach 2.38 m·s^−1^. The peak values of velocity appear in the left of center, and this phenomenon is in accordance with the inclined direction. 

**Figure 6:**
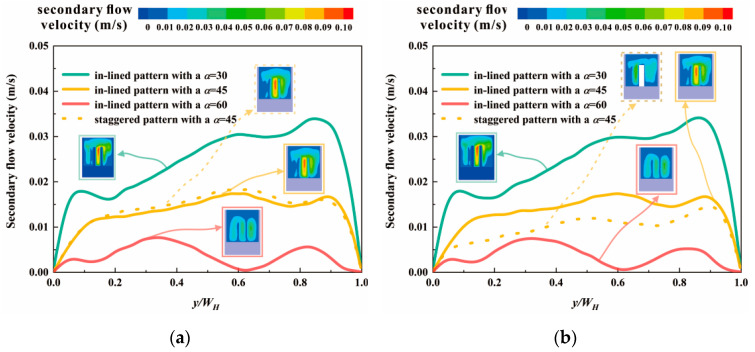
Secondary flow velocities at the plane of (**a**) dimensionless flow length *x*/*L* = 0.257; (**b**) dimensionless flow length *x*/*L* = 0.743.

The addition of pin fins gives rise to secondary flow, which is one significant mechanism of heat transfer enhancement. The secondary flow velocities at the center line of cross sections are plotted in Figure 6, relevant to two planes with a dimensionless flow length *x*/*L* of 0.257 and 0.743, respectively. Compared with *α* = 30° and *α* = 45°, less severe swirl flow generates in the MCHS with pin fins arranged at an angle of 60° to the *y*-axis. In Figure 6a,b, there are similarities between the secondary flow velocity conditions of cases with in-lined pin fins at different planes. In contrast, the secondary flow distributions of the MCHS with staggered pin fins are dissimilar in the planes with *x*/*L* = 0.257 and 0.743, marked in yellow dotted lines. This can be attributed to various numbers of pin fins in one oblique array. The blank area in the contour of secondary flow velocity, as displayed in Figure 6b, is the cross section of pin fin mounted on the substrate. It can be revealed that mixture of fluids close to pin fins scarcely exists. Additionally, disturbance of flow is prone to be enhanced in the center region of cross sections, instead of areas near side wall. Consequently, heat transfer between the mainstream and flow boundary layer near the central zone is facilitated, where fluids absorb more heat.

**Figure 7:**
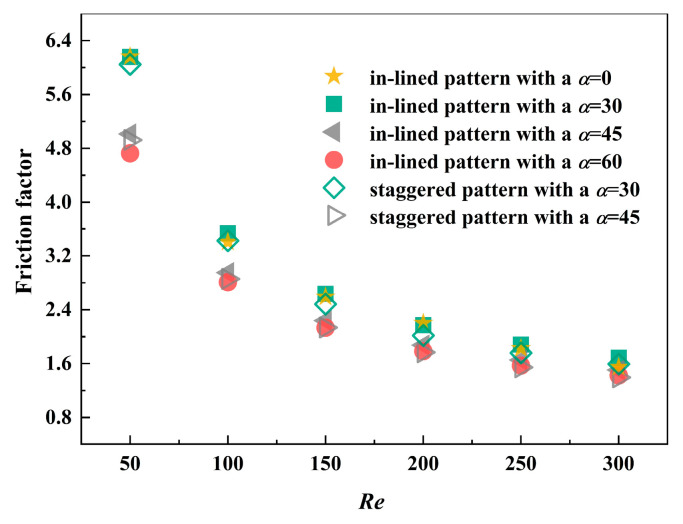
Friction factor of four different MCHS with pin fins.

Compared with pressure drop, friction factor is a better reflection of hydraulic performance, which takes flow velocity into account. From the results illustrated in Figure 7, investigation is conducted in the Reynolds number range of 50 to 300. Typically, thermal performance is improved at the sacrifice of pressure penalty. With the increase of *Re*, friction factor falls rapidly at the beginning and the trend slows down gradually. In the general, the flow resistance in the MCHS with pin fins arranged at an angle of 30° is larger, which is about 1.12~1.24 times that of the MCHS with pin fins arranged at an inclined angle of 45°. The disparity in friction factor of MCHSs with in-lined and staggered pin fins is inconspicuous. Among the cases of four inclined angles, the MCHS with pin fins arranged at an angle of 30° has the largest friction factor, followed by that with an inclined angle of 0°. 

### 3.2. Thermal Performance

**Figure 8:**
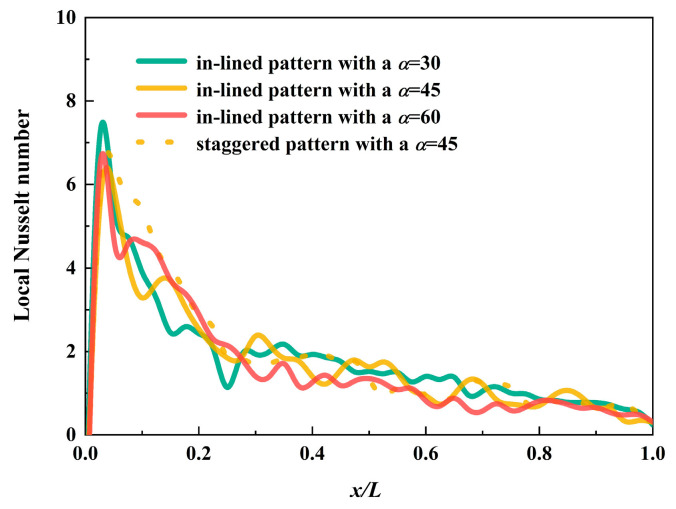
Local Nusselt number of four different MCHS with pin fins.

Local Nusselt number along the flow direction is demonstrated in Figure 8. The inlet effect makes the entrance region an indispensable part especially in miniaturized devices [32]. Four curves representing different arrangement methods and inclined angles have similar pattern, all of which experience a sharp increase when dimensionless flow length is less than 0.05. In the second half of the flow process, the value of local Nusselt number has a tendency to stop rising or falling, indicating a fully developed flow state sooner or later. The results of the MCHS with pin fins arranged at an angle of 30° are slightly beyond that of other cases.

**Figure 9:**
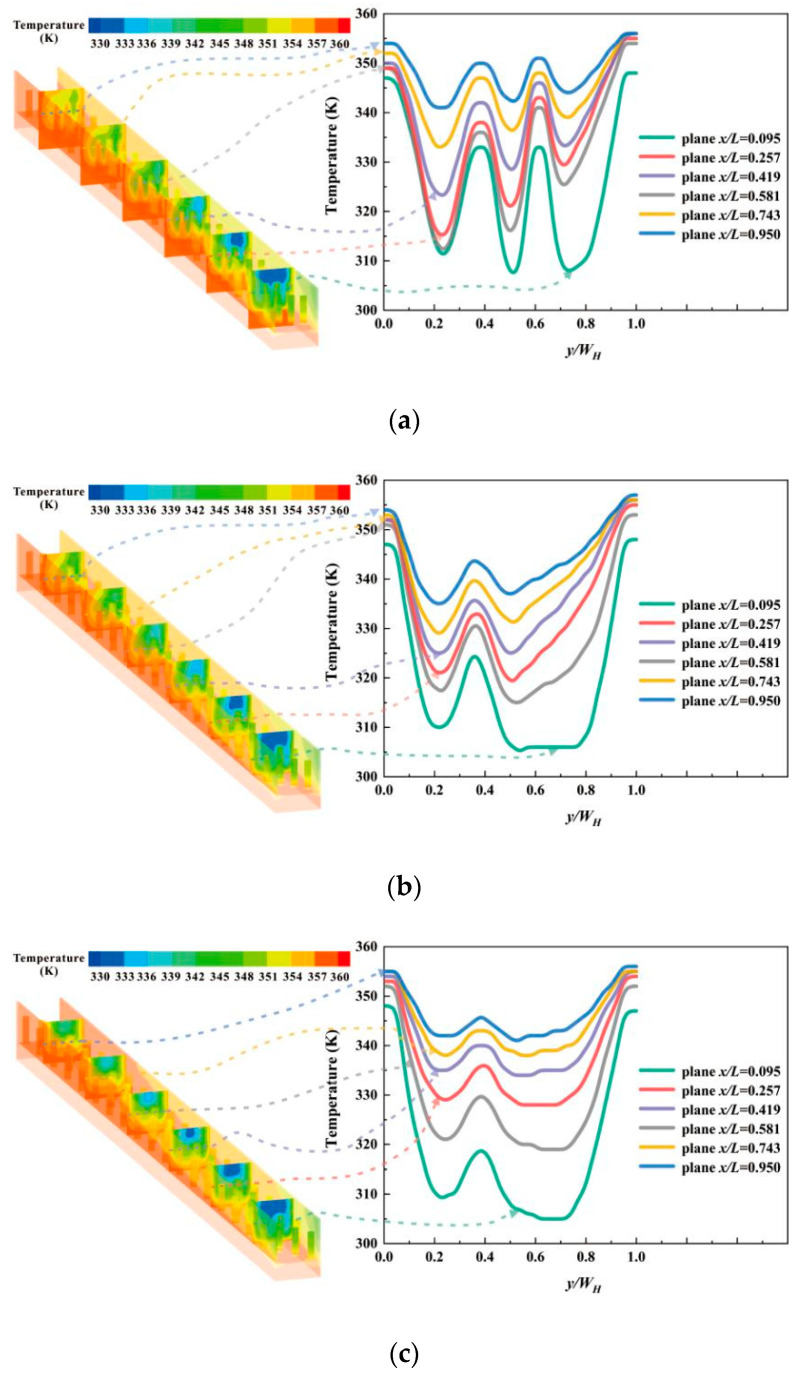

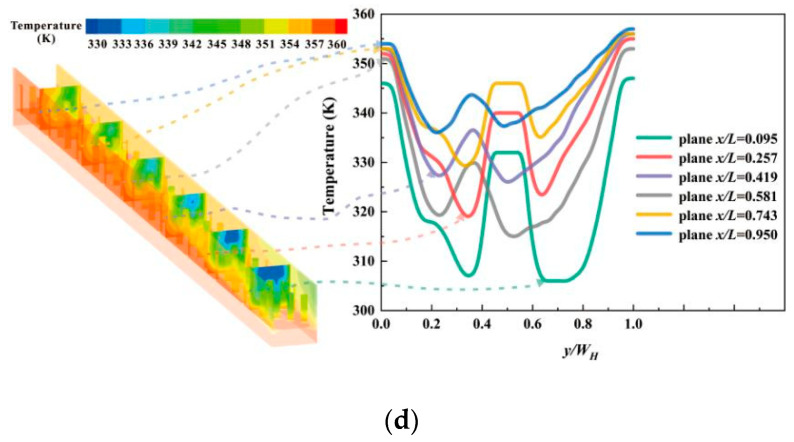
Temperature distributions of the MCHS inserting (**a**) in-lined pin fins with an inclined angle of 30°; (**b**) in-lined pin fins with an inclined angle of 45°; (**c**) in-lined pin fins with an inclined angle of 60°; (**d**) staggered pin fins with an inclined angle of 45°.

The flow of fluid is accompanied with heat transfer from the hotter side to the coolant side. Specific and detailed temperature distribution is displayed in Figure 9. Six planes with the same spacing are selected for comparison, with a dimensionless flow length *x*/*L* = 0.095, 0.257, 0,419, 0.581, 0.743, 0.950, respectively. From the line graphs, the changing situation of temperature in the width direction follows similar law. In the regions near the side walls, the temperature of fluid is close to 355 K, which falls to a certain value rapidly and rises with a relatively low amplitude. The distribution of hotter fluid is consistent with the region of intenser secondary flow, as delineated in Figure 9. When pin fins are arranged in a line with an inclined angle of 30°, there are two eminences in the chart, more than other cases. In other words, the fluid temperature in Figure 9a is higher comparatively, indicating more heat exchanged with the substrate. The general value of temperature in the case with *α* = 60° is lower than that with *α*= 45° when both of their arrangements follow the in-lined pattern. In the staggered pattern, the number of pin fins in one oblique array alters. That is the reason why temperature distributions in Figure 9d have two different rules. Near the oblique array of three pin fins, a wider range of peak value is observed in the center region of the planes. When it comes to the array of pin fins, the variation tendency of temperature is similar to those with in-lined pin fins. Additionally, the positions of crest in the figure move away with the change of pin fin number.

**Figure 10:**
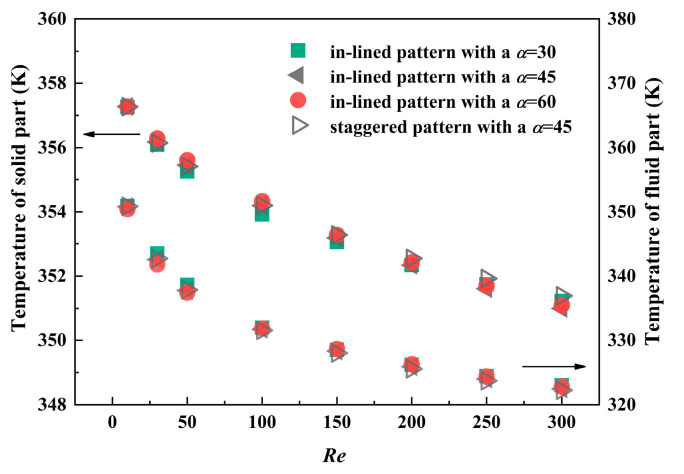
The influence of *Re* on temperature of solid part and fluid part in four different MPFHS.

The variation of Reynolds number also exerts an influence on the temperature condition. Both temperature of solid part *T_a,s_* and that of fluid part *T_a,f_* are demonstrated in Figure 10. As can be seen, the temperature difference between solid and fluid is smaller when inclined angle is 0°, which is an evaluation basis of heat transfer enhancement. With an increase of Reynolds number, the discrepancy between fluid temperature is smaller, especially for *Re* larger than 100, as well as the variance between solid temperature of different models. 

**Figure 11:**
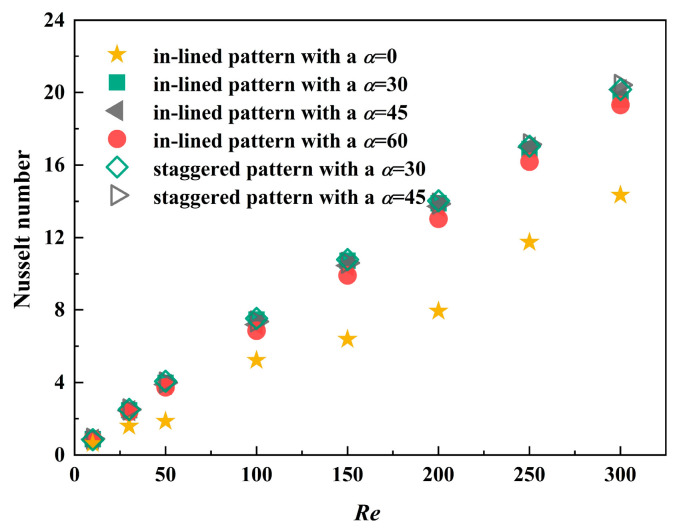
Nusselt number of four different MCHS with pin fins.

Because of slender channels inside, the thickness of flow boundary layer is drastically reduced, which is another major mechanism of heat transfer enhancement. The dimensionless heat transfer coefficient, Nusselt number, is regarded as an evaluation index of thermal performance. From the perspective of entire domain, the MCHS inserting in-lined pin fins with an inclined angle of 30° has little advantage over other conditions in the Reynolds number range of 10 to 200, as depicted in Figure 11. Consistent with the preceding discovery, the way of in-lined or staggered arrangement method has no apparent influence under the circumstance of the same included angle. When pin fins are mounted in an oblique array, the values of *Nu* are reduced by about 3% compared with the staggered arrangement method. There is no direct relationship between the increase of included angle and the enhancement of heat transfer performance. Generally, when fins are arranged in an inclined angle of 0°, the thermal performance of the MPFHS has no advantage over other conditions. Associated with the friction factor in Figure 7, inclined angle plays an important role on the comprehensive performance, with an increase in dimensionless heat transfer coefficient at a little sacrifice of pressure drop. In the case of the MCHS with an inclined angle of 60°, the dimensionless heat transfer coefficient is approximately 94% of the model with staggered pin fins at an inclined angle of 45°.

**Figure 12:**
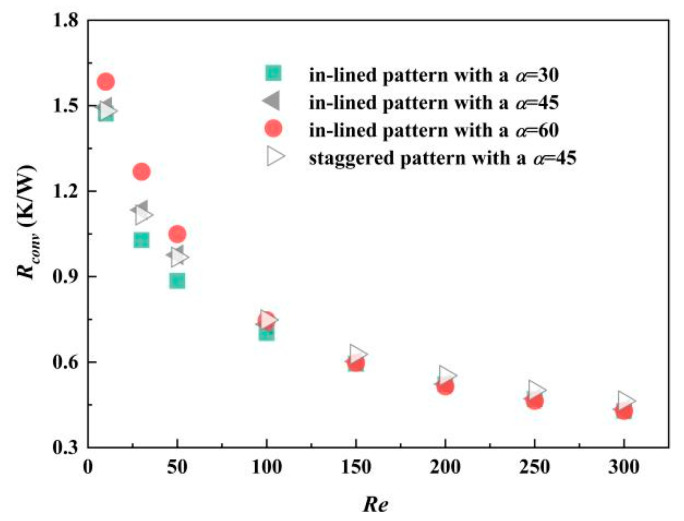
Thermal resistance of four different MCHS with pin fins.

Thermal resistance is the embodiment of convective heat transfer performance combining structural parameters and thermo-hydraulic parameters. Owing to the reduction of flow boundary layer thickness, thermal resistance decreases while heat transfer rate increases. The faster the inlet flow rate is, the smaller the convective thermal resistance is. Accompanied with higher *R_cov_*, more pumping power is in need for coolant flow. When Reynolds number ranges from 100 to 300, the maximum convective thermal resistance is obtained by the MCHS with staggered pin fins at an inclined angle of 45°. Similar to *Nu*, convective thermal resistance *R_cov_* does not increase with the augmentation of inclined angle, which can be inferred from Figure 12. There is a need to mention that inclined angle plays a greater role than arrangement method of pin fins, such as in-lined or staggered pattern.

## 4. Conclusions

In this investigation, the effect of inclined angle and arrangement method of pin fins on thermo-hydraulic performance of MCHS is studied with the help of the finite volume method. In the steady laminar flow with a Reynolds number of 10~300, major conclusions can be summarized as follows:

1. In the MCHS inserting in-lined pin fins with an inclined angle of 30°, intenser secondary flow is generated to facilitate disturbance flow at the sacrifice of a larger friction factor.
2. There is no direct relationship between increase of inclined angle and heat transfer augmentation. The comprehensive performance of the MPFHS with an inclined angle of 0° has no advantage over other conditions.
3. When arrangement method of pin fins changes from in-lined pattern to staggered pattern, the consequent difference in thermo-hydraulic performance is inconspicuous compared with that imposed by the variation of inclined angle.

Deep research on optimum design of MPFHS will be conducted under the same pumping power.

## Nomenclature

*A*	Local area	mm^2^
*A_f_*	Wetted surface area of the MPFHS	mm^2^
*A_s_*	Total base area of MCHS	mm^2^
*C_p_*	Specific heat capacity	J·kg^−1^·K^−1^
*c_p,f_*	Specific heat capacity of fluid	J·kg^−1^·K^−1^
*D_h_*	Hydraulic diameter	mm
*d_f_*	Diameter of fin	mm
*f*	Friction factor	
*h_i,x_*	Local heat transfer coefficient	W·m^−2^·K^−1^
*H_c_*	Height of microchannel	μm
*H_f_*	Height of fin	μm
*H_s_*	Height of substrate	μm
*k_f_*	Thermal conductivity of fluid	W·m^−2^·K^−1^
*k_s_*	Thermal conductivity of solid	W·m^−2^·K^−1^
*L*	Length of the microchannel	mm
$\overset{\cdot }{m}$	Mass flux rate of water	kg·s^−1^
*Nu*	Nusselt number	
*Nu_x_*	Local Nusselt number	
*P*	Pressure	Pa
*Q*	Volumetric flow rate of fluid	mL/min
*q*	Local heat flux	W·m^−2^
*R_conv_*	Average thermal resistance of convection	K/W
*Re*	Reynolds number	
*T_a,f_*	Volume-weighted average temperature of fluid part	K
*T_a,s_*	Volume-weighted average temperature of solid part	K
*T_f_*	Temperature of fluid	K
*T_i_*	Inlet temperature of fluid	K
*T_o_*	Inlet temperature of fluid	K
*T_s_*	Temperature of solid	K
*u*	Flow velocity	m·s^−1^
*u_max_*	Maximum flow velocity	m·s^−1^
$\vec{V}$	Velocity vector	m·s^−1^
*V_f_*	Fluid volume of the MPFHS	mm^3^
*v_yz_*	Secondary flow velocity	m·s^−1^
*W_H_*	Width of single unit cell in MPFHS	μm
**Greek symbols**		
*α*	Inclined angle	^°^
*μ*	Viscosity	Pa∙s
*ρ*	Density	kg·m^−3^
Δ*P*	Pressure difference	Pa
